# Integra Dermal Regeneration Template in Reconstruction of Primary Oncologic Defects in the Lower Extremities: A Case Series

**DOI:** 10.1177/22925503251336254

**Published:** 2025-05-08

**Authors:** Katie Ross, Nicholas Zinck, David Wilson, Jack Rasmussen, Michael Biddulph, Jason Williams

**Affiliations:** 1 Department of Surgery, Division of Plastic Surgery, 3688 Dalhousie University, Halifax, Nova Scotia, Canada; 2 Faculty of Medicine, 3688 Dalhousie University, Halifax, Nova Scotia, Canada; 3 Department of Surgery, Division of Orthopedic Surgery, 3688 Dalhousie University, Halifax, Nova Scotia, Canada; 4 Department of Critical Care Medicine, 3688 Dalhousie University, Halifax, Nova Scotia, Canada

**Keywords:** integra dermal regeneration template, integra, reconstruction, oncology, extremity, matrice de régénération dermique Integra, reconstruction, oncologie, extrémité

## Abstract

This article presents a case series of 4 patients who underwent primary reconstruction of oncologic defects in the lower extremities using Integra Dermal Regeneration Template (IDRT). The patients had either primary or recurrent tumors, which resulted in exposure of deep underlying structures including tendon, nerve, muscle, and bone. IDRT was selected to manage these defects due to the uncertain malignant potential of tissue margins and its ability to facilitate later margin revision without sacrificing tissue. The use of IDRT allowed for the growth of a neodermis that supported subsequent split-thickness skin grafting in all cases. Additionally, for those with positive margins, surgical revision and skin graft application was able to be performed in a single procedure, maximizing operating room use and patient convenience. This case series highlights the potential of IDRT in managing complex oncologic defects in the lower extremities, expanding options for reconstructive surgeons. Key findings: (1) IDRT is a viable option for reconstruction in oncologic resections exposing deep structures. (2) In cases with unknown malignant potential of tissue margins, use of IDRT can allow for revision of positive margins without sacrificing graft or flap tissue. (3) Negative pressure wound therapy is an important adjunct in achieving a favorable neodermis for acceptance of a spilt thickness skin graft.

## Résumé

Cet article présente une série de quatre cas de patients ayant subi une reconstruction primaire de manques de tissus d’origine oncologique au niveau des membres inférieurs en utilisant la matrice de régénération dermique Integra (IDRT). Les patients avaient eu des tumeurs primitives ou récidivantes ayant abouti à l’exposition de structures sous-jacentes profondes, incluant tendons, nerfs, muscles et os. L’IDRT a été choisi pour gérer ces manques en raison du potentiel malin indéterminé des marges tissulaires et de sa capacité à faciliter une révision ultérieure de la marge sans sacrifier de tissus. L’utilisation de l’IDRT a permis, dans tous les cas, la croissance d’un néoderme qui a soutenu un greffon subséquent dermoépidermique. De plus, pour les patients ayant des marges positives, une révision chirurgicale et l’application d’une greffe cutanée ont pu être réalisées au cours d’une seule procédure, permettant d’exploiter au maximum la salle d’opération pour la commodité du patient. Cette série de cas souligne le potentiel de l’IDRT dans la gestion des manques complexes des membres inférieurs d’origine oncologique, élargissant les options à la disposition des chirurgiens plasticiens.

Principales constatations : 1. La matrice de régénération dermique Integra (IDRT) constitue une option viable pour la reconstruction en cas de résections oncologiques exposant des structures profondes. 2. En cas de potentiel malin inconnu des marges tissulaires, l’utilisation de l’IDRT rend possible la révision de marges positives sans sacrifier la greffe ou le volet tissulaire. 3. Un traitement de plaie par pression négative est un important appoint pour l’obtention d’un néoderme favorable à la prise d’une greffe cutanée dermoépidermique.

When faced with a challenging defect, the reconstructive surgeon has various closure techniques and materials available, ranging from the simple to the very complex. One unique option is Integra Dermal Regeneration Template (IDRT).^1,2^

The use of IDRT in reconstructive surgery has significantly expanded over the past decade with case reports describing its use in burn injuries, flap donor defects, and traumatic injuries.^1,3–12^ In some of these cases, the defects exposed deeper tissues such as muscle, tendon, and bone; despite this, positive outcomes were achieved.^5,11^ Traditionally, deep defects have required free autologous tissue transfer. While these remain a critical part of the reconstructive ladder, they are not without obstacles including flap failure, donor site morbidity, long operative times, and the need for a prolonged hospital stays for flap monitoring. Although there has been increased use of IDRT in recent years, there is a lack of literature assessing its benefit for reconstruction of oncologic defects exposing deep tissues in the lower extremities.

This article describes 4 cases in which IDRT has been used in the primary reconstruction of deep oncologic defects of the lower extremities with the aim of demonstrating the efficacy in this specific context.

**Figure 1.**
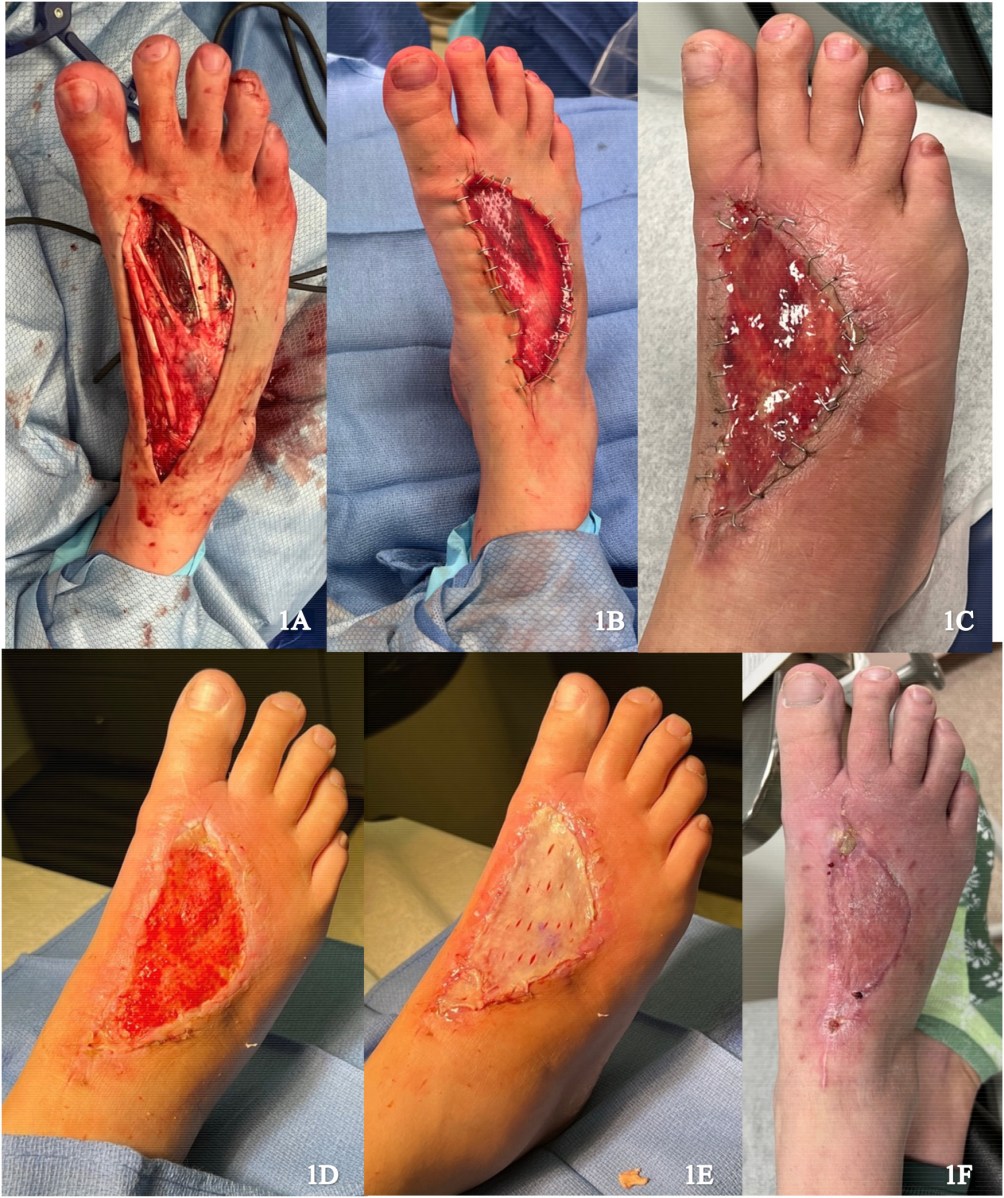
(A) Resection of the tumor; (B) Integra placement; (C) Vascularized Integra after 3 weeks; (D) Removal of Integra silicone layer; (E) Initial skin graft placement; and (F) Healing skin graft.

A 41-year-old female presented with recurrence of a previously excised pleomorphic hyalinizing angiectatic tumor on the dorsum of the right foot. Re-excision was performed with circumferential dissection of the tumor, raising fascia and paratenon of extensor hallucis longus, with exposure of deep tissues (Figure 1A). Local skin advancement was performed to reduce the wound size and IDRT was applied (Figure 1B and C). The wound was sealed with a negative pressure wound therapy (NPWT). Formation of a neodermis was achieved 3 weeks following IDRT application (Figure 1D). Under local anesthetic, a spilt thickness skin graft (STSG) (0.0012 inches) measuring 8 × 10 cm was harvested from the right proximal thigh and secured to the wound after removal of the silicone layer (Figure 1E). NPWT was applied and there was complete graft take at 1 week. No complications were noted on follow up (Figure 1F).

**Figure 2.**
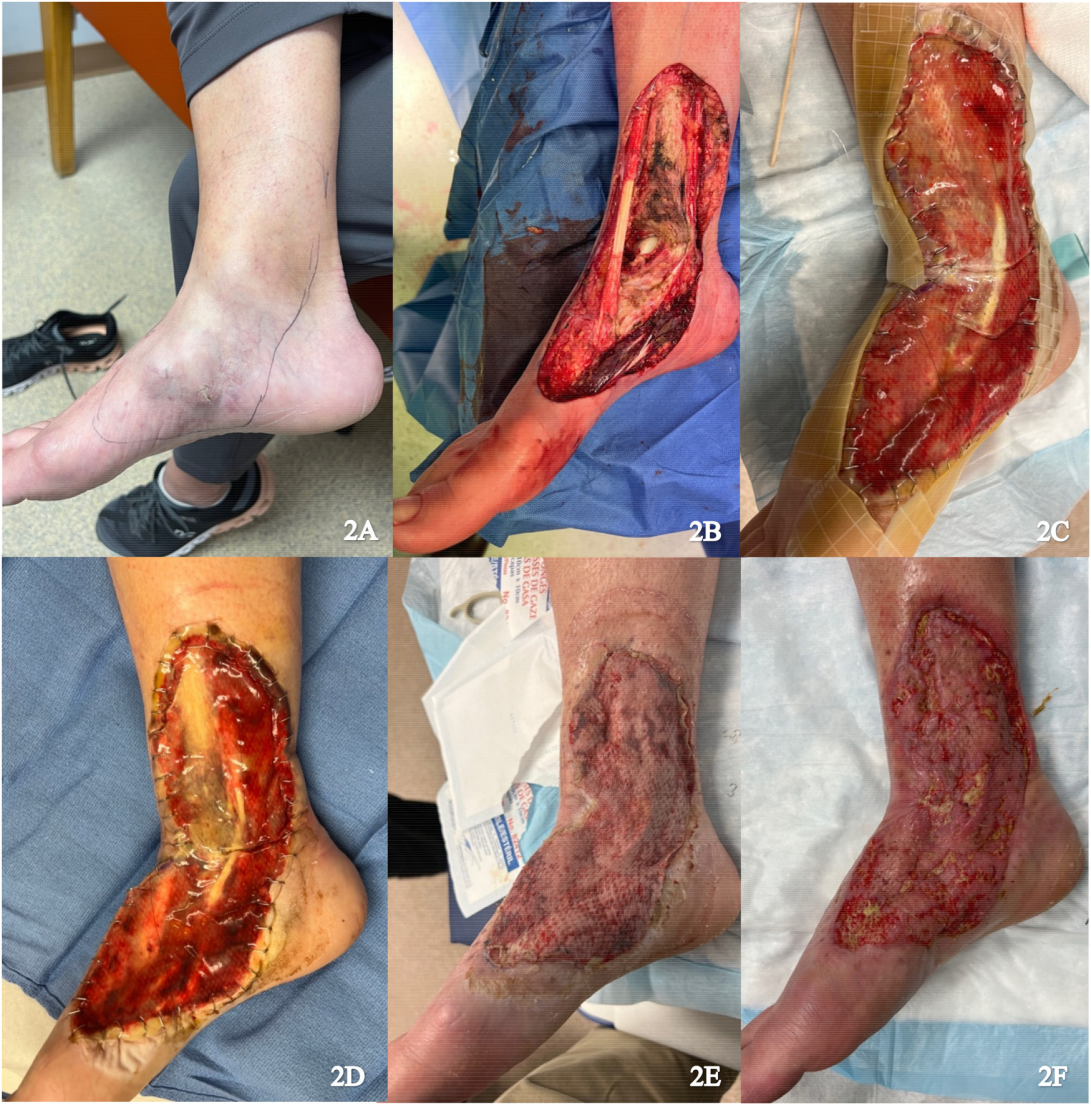
(A) Preoperative image of right foot; (B) Resection of the tumor; (C) Integra and NPWT placement; (D) Vascularized Integra; (E) Skin graft after 1 week; and (F) Healing skin graft. Abbreviation: NPWT, negative pressure wound therapy**.**

A 56-year-old female presented with multifocal myxoinflammatory fibroblastic sarcoma of the medial right foot (Figure 2A). Resection was performed with neurolysis of the posterior tibial nerve and deep peroneal nerve, dissection of the posterior and anterior tibial arteries, and dissection of fascia and paratenon from surrounding structures (Figure 2B). Local tissue advancement was performed followed by IDRT application (Figure 2B and C). The wound was sealed with NPWT.

Approximately 2 weeks after the procedure, histopathology confirmed a positive posterior margin requiring revision. IDRT was continued as the patient indicated a strong preference to avoid flap surgery due to the potential donor site morbidity and the anticipated bulk of a flap that could interfere with wearing shoes. Revision of the positive margin, removal of the silicone layer, and application of an STSG (0.0012 inches) from the right lateral thigh was performed 5 weeks after the initial procedure (Figure 2E). The final wound size measured appropriately 18 × 8 cm. NPWT was applied and there was successful graft take at 1-week follow up (Figure 2F).

**Figure 3.**
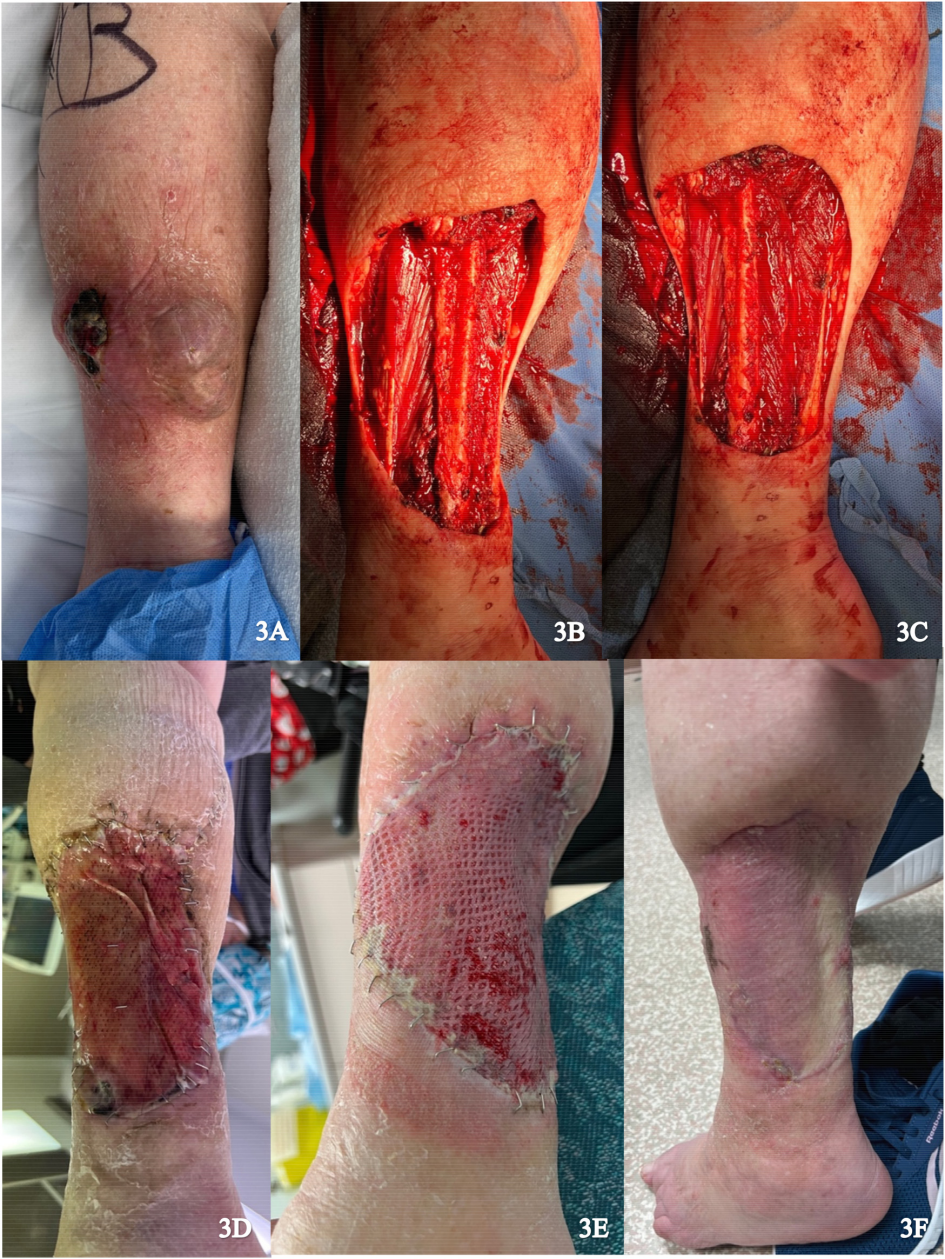
(A) Preoperative image of posterior left lower leg; (B) and (C) Resection of the tumor; (D) Vascularized Integra; (E) Skin graft placement; and (F) Healed skin graft.

A 92-year-old female presented with a large myxofibrosarcoma on the posterior aspect of the left lower leg (Figure 3A). Excision of the tumor involved resection of portions of the medial gastrocnemius and soleus muscles. In addition, periosteum was dissected from the fibula and the peroneal nerve was exposed (Figure 3B and C). Muscle flaps were used to cover the exposed fibula and peroneal nerve and skin flaps were advanced to reduce wound size. IDRT was applied to the wound and sealed with NPWT (Figure 3D). Complete revascularization and formation of neodermis was achieved after 3 weeks, with exception of a small distal area. An STSG (0.0012 inches) was harvested from the left proximal lateral thigh and applied over the 10 × 15 cm wound after removal of the IDRT silicone layer (Figure 3E). NPWT was reapplied and there was complete graft take after 2 weeks (Figure 3F).

**Figure 4.**
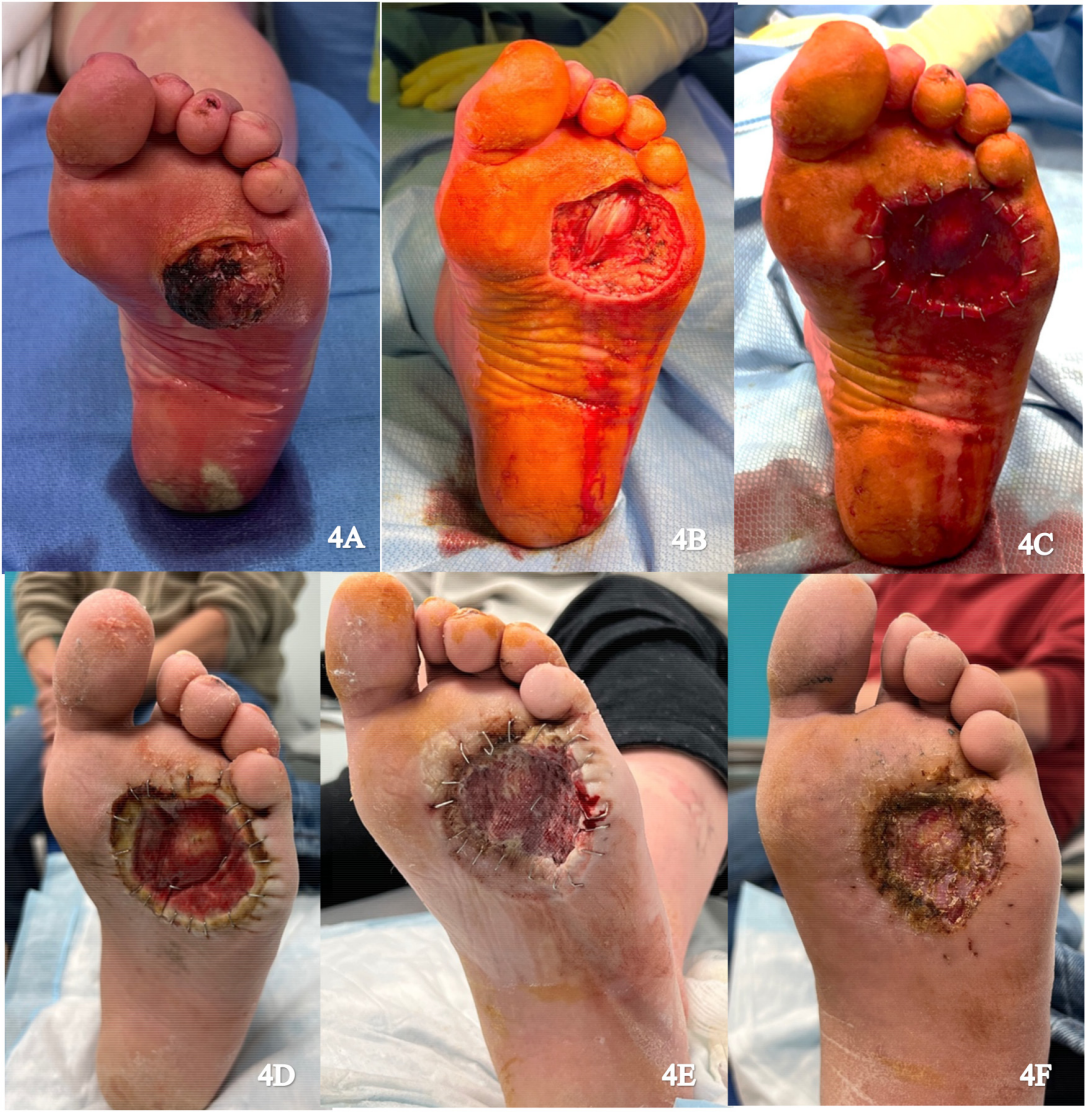
(A) Preoperative image of the left foot; (B) Resection of the tumor; (C) Integra placement; (D) Vascularized Integra; (E) Skin graft placement; and (F) Healing skin graft.

A 49-year-old male presented with a verrucous squamous cell carcinoma on the plantar aspect of the left foot (Figure 4A). Excision of the tumor resulted in exposure of the flexor tendons over the metatarsophalangeal joint capsule (Figure 4B). An IDRT sheet measuring 6 × 6 cm was applied over the wound (Figure 4C). The wound was sealed with an NPWT dressing and reapplied weekly (Figure 4D). Histopathology confirmed a close positive lateral margin. Four weeks later, the margin was revised and an STSG from the left thigh was applied to the neodermis following removal of the IDRT silicone layer (Figure 4E). NPWT was reapplied and there was 100% graft take after 1 week with no complications on follow up (Figure 4F).

We report a case series of 4 patients managed for reconstruction of primary oncologic defects of the lower extremities using IDRT. The patients range from 41 to 92 years of age with either primary growth or local recurrence of tumors in a variety of regions of the lower extremities.

In each of the cases presented, initial reconstruction utilizing IDRT was selected partly due to the unknown malignant potential of tissue margins. In contrast to a primary skin flap closure, initial management with IDRT allowed for later revision of positive margins without sacrificing graft or flap tissue, while at the same time allowing for growth of a neodermis capable of accepting an STSG. In each case, revision was able to be performed in the same procedure as final closure via skin grafting, minimizing patient inconvenience and use of operating room time.

The timing for removing the silicone layer is typically between 3 to 4 weeks, based on neodermis formation.^13^ In our case series, 3 patients were ready for skin grafting within this time frame, whereas 1 patient required prolonged grafting until an appropriate wound bed had formed. When managing these patients, our experience showed that the NPWT dressing is an important adjunct in achieving good results. NPWT dressings were used throughout the entire process and were changed at weekly appointments which also allowed for frequent assessment of neodermis formation. Splinting was not routinely required but was considered in cases where joint motion could interfere with graft adherence and cause shearing.

In all cases, tumor excision resulted in exposure of deep structures. NPWT has been used alone to manage wounds by removing excess fluid, enhancing angiogenesis, and fibroblast migration.^14^ While this process can facilitate formation of granulation tissue matrix, it typically is not be placed it directly on exposed tendon, bone, or nerve.^15,16^ In these situations, using a dermal regeneration template like Integra can provide a layer that provides protection and is effective in regenerating tissue that can accept a delayed skin graft. In our cases, combining the 2 was considered superior to using either alone for complex wounds with exposed deep structures.

IDRT is a commercially available product with significant costs, varying based on size. Institutions should account for this when considering IDRT in their reconstructive algorithm. Handling and storage guidelines recommend maintaining the product in a temperature-controlled environment and ensuring proper hydration before application.^17^

The reconstructive surgeon has many considerations when faced with challenging defects. The choice of reconstructive technique will depend on the nature of the defect, available donor sites, and patient factors. With the successful use of IDRT in complex oncologic defects, we identify another viable option to add to the list of available techniques.

## Supplemental Material

sj-docx-1-psg-10.1177_22925503251336254 - Supplemental material for Integra Dermal Regeneration Template in Reconstruction of Primary Oncologic Defects in the Lower Extremities: A Case Series Supplemental material, sj-docx-1-psg-10.1177_22925503251336254 for Integra Dermal Regeneration Template in Reconstruction of Primary Oncologic Defects in the Lower Extremities: A Case Series by Katie Ross, Nicholas Zinck, David Wilson, Jack Rasmussen, Michael Biddulph and Jason Williams in Plastic Surgery
